# Ulcerative Colitis Activity Presenting as Fever of Unknown Origin, in a Patient with Longstanding Disease under Continuous Treatment with Mesalazine

**DOI:** 10.1155/2016/4396256

**Published:** 2016-07-18

**Authors:** Panagiota Voukelatou, Elissavet Sfendouraki, Theodoros Karianos, Sofia Saranti, Dimitrios Tsitsimelis, Ioannis Vrettos, Andreas Kalliakmanis

**Affiliations:** ^1^2nd Department of Internal Medicine, General Oncological Hospital of Kifissia “Ag. Anargyroi”, Kifissia, 145 64 Athens, Greece; ^2^Department of Nuclear Medicine, General Oncological Hospital of Kifissia “Ag. Anargyroi”, Kifissia, 145 64 Athens, Greece; ^3^Department of Radiology, General Oncological Hospital of Kifissia “Ag. Anargyroi”, Kifissia, 145 64 Athens, Greece

## Abstract

*Background*. The presence of ulcerative colitis (UC) with no bowel symptoms, as fever of unknown origin (FUO), is uncommon.* Objective*. To describe the case of an 80-year-old woman who presented with fever, with a history of UC under treatment with mesalazine.* Case Presentation*. She was admitted due to fever lasting for 12 days with no associated symptoms. Seven years earlier, she was diagnosed with UC. After an extended workup for FUO that failed to reach the diagnosis, she underwent a gallium-67 scintigraphy. This revealed a persistent diffuse concentration of gallium-67 in the ascending colon at 24-hour imaging that remained stable at 48- and 72-hour imaging without any topographic change after the use of laxatives. Considering the results and in the absence of another diagnosis, the patient was treated with 30 mg prednisone daily and mesalazine, as treatment of active UC. Subsequently, the patient's condition improved markedly and the fever retreated. One month later, she was reevaluated with a gallium-67 scintigraphy with total absence of gallium-67 concentration in the ascending colon.* Conclusion*. UC activity must be included in the differential diagnosis of FUO in patients with longstanding disease, since fever may present alone, with no other manifestations.

## 1. Background

Fever of unknown origin (FUO) constitutes a diagnostic challenge for physicians. The cause of fever in many cases remains elusive despite the technological progress [1] and a definitive diagnosis is not eventually reached in a proportion as high as 51% of cases, according to one of the latest published series [2]. However, the cause of fever is mainly attributed to a common disease with an atypical presentation rather than a less common disease [3].

The diagnostic workup for FUO often involves numerous invasive and noninvasive procedures. However, sometimes it fails to explain the cause of fever [4].

When the initial diagnostic evaluation fails to determine the cause of fever, additional, and in some cases individualized, investigation is required [3–5].

Among the diagnostic techniques involved in the documentation of FUO, nuclear metabolic procedures (NMP) are highly attractive due to the following reasons: (1) NMP detects the infection earlier than the diagnostic anatomical techniques according to the rules stating that “metabolic changes precede anatomic changes and metabolic changes can be shown in the absence of anatomic changes”; (2) NMP involve the entire body. Several radiopharmaceuticals have been used in FUO with a large diversity in diagnostic accuracy and sensitivity according to their mixed tumoral and inflammatory biological characteristics and the opportunistic or not opportunistic character of the diseases. Labeled leukocytes, gallium-67, and the most recent F-18 FDG are of special interest [6, 7].

In this report, we describe the case of an 80-year-old woman with ulcerative colitis activity who underwent an extended workup for FUO that failed to reach diagnosis but was eventually diagnosed by gallium-67 scintigraphy.

## 2. Case Presentation

An 80-year-old woman was admitted with reported fever that started 12 days before with no associated symptoms. According to her medical history, she was diagnosed with a myelodysplastic syndrome (MDS) 8 years ago, which was effectively managed with erythropoietin. Seven years earlier, she was diagnosed with ulcerative colitis, treated with mesalazine 800 mg twice daily. Finally, a tuberculosis (TB) infection was reported more than 40 years before. A former urine examination had revealed a urinary tract infection (urinary culture with the presence of* E. coli* > 10^5^ CFU susceptible to almost all antibiotics). She received amoxicillin/clavulanic acid for a week and subsequently ciprofloxacin, one week prior to her presentation to the emergency department, but fever failed to settle.

At presentation, her body temperature was 38.5°C and she was hemodynamically stable. She reported no cough, dyspnea, abdominal pain, diarrhea, or dysuria. She had no clinical signs indicating a respiratory, urinary, or abdominal infection while a systolic aortic valve murmur was present. Her ECG revealed a 1st-degree AV block and her chest X-ray showed a cavity at the right upper lung field. Her tests revealed anemia (HCT = 23%, HGB = 7.2 mg/dL), a low WBC count, an elevated ESR (>100 mm/h), and CRP = 71.4 mg/L (0.0–5.0 mg/L); thus, she received 1 RBC unit for treating anemia. Fever was initially considered as a symptom of a urinary tract infection, which did not respond to the antibiotic treatment per os, and therefore she was treated with intravenous meropenem. At the same time, the patient received multiple blood transfusions, due to the fact that she was not responding to erythropoietin.

**Figure 1:**
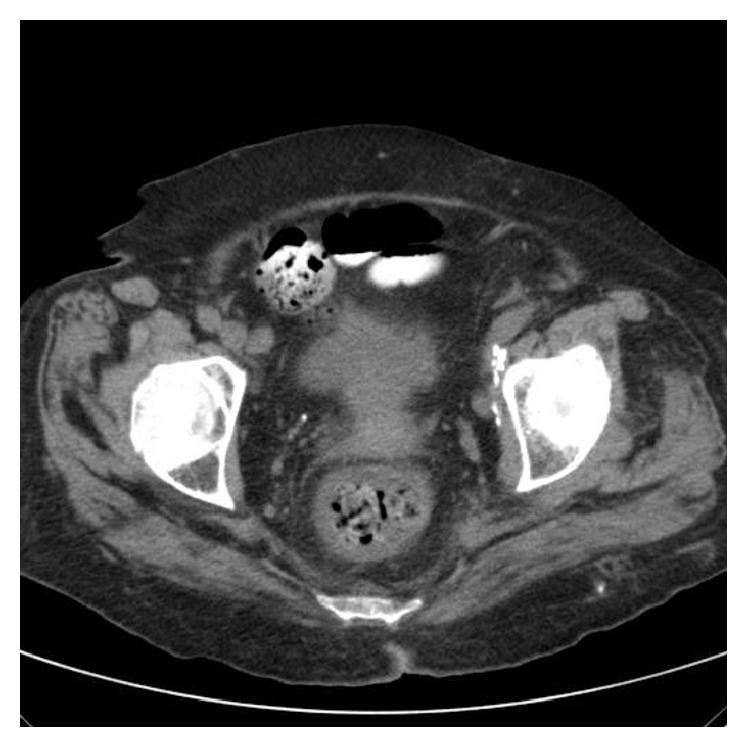
Edema of the rectum.

Since fever was persisting even after antibiotic treatment, simultaneously further investigation was deemed necessary. The patient had a CT scanning of the chest and the abdomen. The chest scanning revealed a cavity at the right upper lung field, which was consistent with the history of the old TB infection and the chest X-ray findings. The abdominal scan did not reveal any significant findings apart from the presence of edema at the rectum (Figure 1).

Subsequently, the patient had a colonoscopy, which revealed a thickened fold of rectum mucosa and diverticulosis of the sigmoid with redness of the surrounding mucosa. The patient was treated for 12 days with meropenem before the colonoscopy and diverticulosis was excluded as the cause of pyrexia since her fever did not settle. However, the coexistence of diverticulitis in remission could not be excluded. A few days later, the biopsies from colonoscopy revealed a mild chronic, unspecific inflammation of the sigmoid and the rectum.

Awaiting the results of the biopsies, the patient had a gastroscopy, which only revealed a mild gastritis. The transthoracic cardiac ultrasound confirmed a mild aortic valve stenosis with no other findings indicating endocarditis; thus, we did not proceed to a transoesophageal echocardiogram (multiple blood cultures were all negative). A myelogramconfirmed the MDS, RARS type. Moreover, a full laboratory examination of the immune system was conducted (ANA, anti-dsDNA, anti-ENA, anti-RNP, anti-Ro, anti-La, anti-sm, C-ANCA, P-ANCA, anti-MPO, anti-PR3, and anti-CCP) as well as serum protein electrophoresis, right temporal artery biopsy, and investigation for possible relapse of the old TB infection (Mantoux, urine, gastric fluid, sputum and bone marrow Ziehl-Neelsen stain, and culture for *β*-Koch). Pending the results, the patient was discharged with prescription for paracetamol to treat the fever.

**Figure 2:**
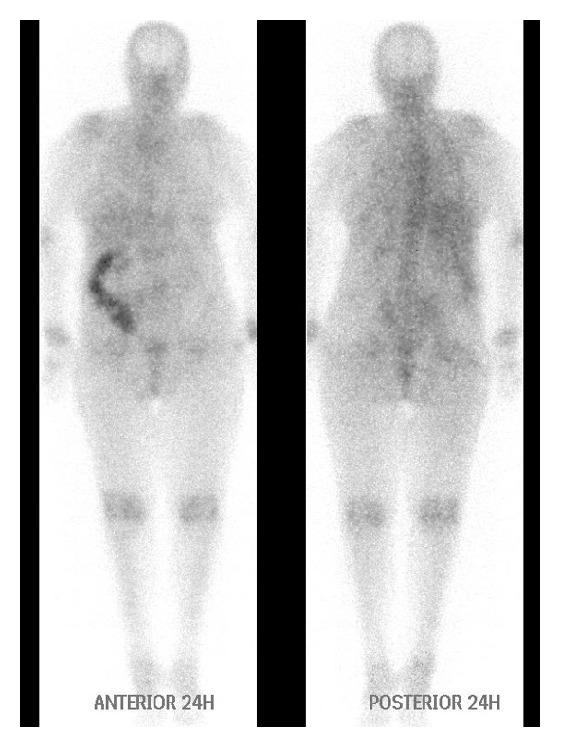
The gallium-67 scintigraphy revealed persistent diffuse concentration of the gallium in the ascending colon at 24-hour imaging. Gallium-67 excreted into the bowel and laxatives are essential for the differential diagnosis between true positive increased bowel activity and false positive uptake due to bowel content.

A few days later, the patient was readmitted for a blood transfusion and she was still febrile. The results of the immunologic tests were inconclusive {ANA (−), anti-ENA (+), and anti-RNP (+)}; the serum protein electrophoresis and the temporal artery biopsy were negative and so were the tests for the reactivation of the old TB infection. The patient was evaluated by a rheumatologist who recommended treatment with 20 mg of prednisone daily. After 21 days, the patient remained febrile and thus underwent a gallium-67 scintigraphy searching for a hidden focus of inflammation. The gallium-67 scintigraphy revealed persistent diffuse concentration of the gallium in the ascending colon (Figure 2) at 24-hour imaging, in discordance with the colonoscopy (no signs of active inflammation at the ascending colon).

**Figure 3:**
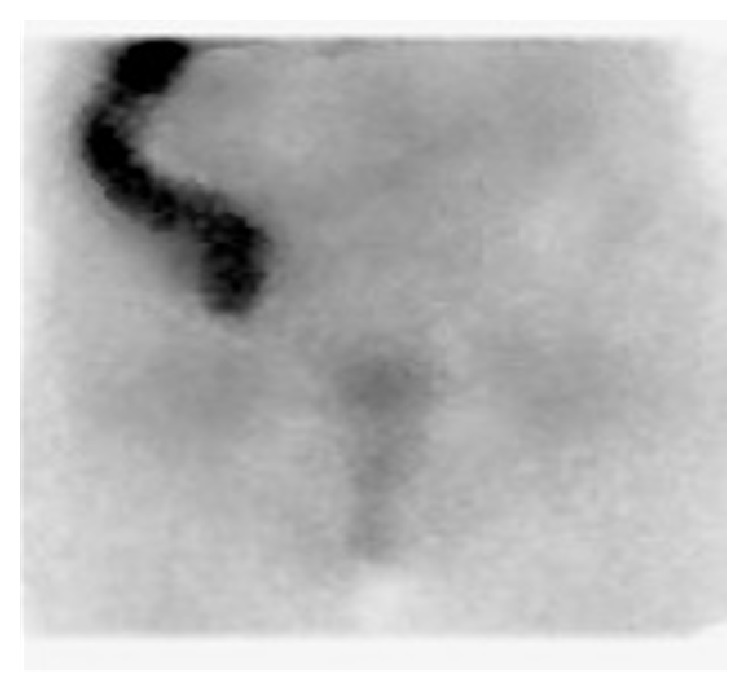
After the use of laxatives, the gallium-67 diffuse uptake of the ascending colon not only remained stable but rather strengthened without any topographic change at the repeated 72-hour imaging, indicating active inflammation.

The gallium-67 diffuse uptake of the ascending colon remained stable at the repeated 48- and 72-hour imaging without any topographic change after the use of laxatives (Figure 3).

**Figure 4:**
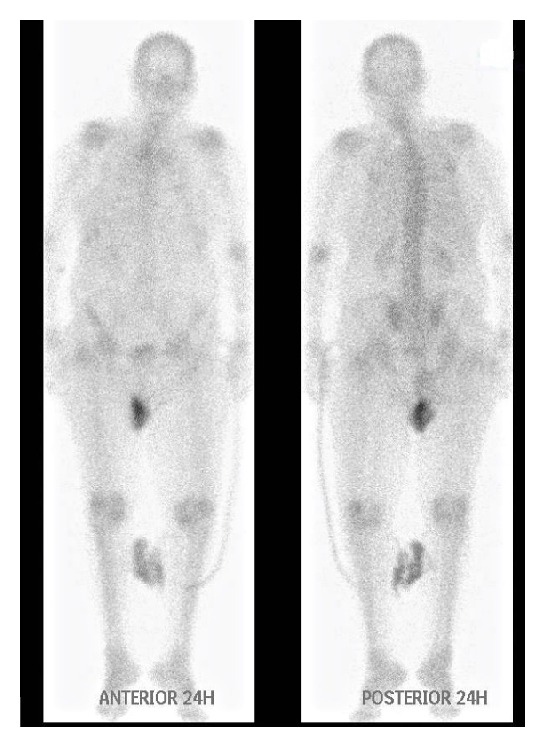
One month later and after appropriate treatment there was a total absence of gallium-67 concentration in the ascending colon.

Considering the results of gallium-67 scintigraphy, colonoscopy, biopsies of rectum and sigmoid, and the absence of another diagnosis, the patient was started on 30 mg of prednisone daily with mesalazine, as treatment for active ulcerative colitis. At the same time, she was receiving isoniazid and rifampicin as prophylaxis for the old TB infection. Subsequently, the patient's condition improved markedly, the fever retreated, and the need for blood transfusions also substantially decreased. The patient was discharged. One month later, she was reevaluated with a gallium-67 scintigraphy, which was notably different, with total absence of gallium-67 concentration in the ascending colon (Figure 4).

The cortisone dosage was decreased and close monitoring of the patient continued on an outpatient basis.

## 3. Discussion 

Ulcerative colitis is a chronic inflammatory bowel disease and its most common feature is the presence of blood and mucus mixed with stool accompanied by cramping in the lower abdomen. Fever is present in 40% of patients at the time of presentation. Usually it is chronic and low grade and may remain unrecognized [8].

More than 200 causes of FUO have been reported [4] and inflammatory bowel disease is among them [3, 9, 10].

In a hospital based study of 120 patients with ulcerative colitis in North India, 4 of them presented with FUO [11]. Moreover, in a prospective multicenter study of 154 patients with FUO in Turkey, aiming to determine the spectrum of diseases causing FUO, one patient was diagnosed with ulcerative colitis [12]. Likewise, in a retrospective chart review of 98 cases with FUO in a 13-year period, Moawad et al. reported 3 cases with ulcerative colitis [9]. Unfortunately, a detailed report of the clinical profile of these patients is unavailable.

On the other hand, Tabata et al. reported a case of a 40-year-old man with ulcerative colitis who initially presented with fever of unknown origin and remained undiagnosed, until bloody stool and abdominal pain came up during the course of his hospitalization [13].

Moreover, Esiyok et al. reported a case of a 61-year-old male patient with fever, sweating, fatigue, and abdominal pain with no other abdominal symptoms, who underwent a colonoscopy as part of an extended workup for FUO. Ulcerative colitis was determined as the fever cause, based on the endoscopic findings and the biopsy [14].

There is also one published case of a 71-year-old woman with a history of ulcerative colitis who was admitted with FUO with no bowel symptoms, thus notably similar to the case we are presenting. The patient was submitted to an extended workup, including positron emission tomography (PET). Pet scanning revealed foci of active inflammation in the ascending and the sigmoid colon in consistence with the history of inflammatory bowel disease. Therefore, ulcerative colitis was considered as the cause of the fever [15].

Among NPM, labeled leukocytes scintigraphy is the method of choice for the detection of ulcerative colitis with 111In-labeled granulocytes totally replaced by ^99m^Tc-HMPAO-labeled granulocytes [16, 17] while positron emission tomography (PET) scintigraphy is a newer challenging nuclear modality with fulfilling sensitivity and specificity [18, 19]. Other radiopharmaceuticals as the tumor seeking labeled agent ^99m^Tc-[V]-DMSA have been also used [20]. For all the above nuclear studies, a persistent diffuse pattern is demonstrated with a major role of laxatives for the differential diagnosis between true positive increased bowel activity and false positive uptake due to bowel content. Gallium-67 scintigraphy is not the first choice as the ideal radiopharmaceutical should not be excreted into the bowel because diffuse or foci uptake could be missed.

We performed a gallium-67 scintigraphy for our patient—as it was at that time the only notably available radiopharmaceutical—with special attention to the bowel excretion limitations.

A cause of concern regarding the diagnosis of ulcerative colitis activity, as the cause of fever in our patient, was the fact that she did not have inflammatory involvement of the rectum according to the gallium-67 scintigraphy, although the biopsy results showed mild, chronic, unspecific inflammation of the rectum. However, several studies have shown that greater disease activity in proximal rather than distal parts of the colon, as well as absence of rectal involvement, may occur during the natural history of treated and longstanding ulcerative colitis [21, 22]. Atypical presentations of left sided inflammation of the colon with cecal or appendiceal involvement can be observed and the affected sites are separated by apparently uninvolved mucosa [23].

Another cause of concern was the fact that our patient had an endoscopically normal ascending colon, in discordance with the results of gallium-67 scintigraphy. In general, the microscopic pattern of ulcerative colitis activity correlates well with endoscopic features of severity, although microscopic features may exist in endoscopically inactive disease [24]. Furthermore, abnormal colorectal histology was found in 14.5% of 447 patients with diarrhea and in 11% of 155 patients without diarrhea, according to biopsies taken from an endoscopically normal large bowel. In one case, the distorted mucosal histological pattern was consistent with inflammatory bowel disease. Moreover, Elliot et al. reported that, among 151 patients with endoscopically normal or near normal colorectal mucosa, 14% (8 patients) had abnormal colorectal histology and two of them had histological findings indicating inflammatory bowel disease, ulcerative colitis type [25].

A third cause of concern was the fact that gallium-67 scintigraphy is considered less effective for the detection of intra-abdominal causes of FUO because of gallium's physiologic excretion in the gut [26]. Nevertheless, when gallium-67 scintigraphy was performed in patients with ulcerative colitis, with concern to the bowel excretion and use of laxatives, there was a good correlation between the regional uptake of gallium-67 and the extent and activity of the disease. Moreover, the scans were positive during acute exacerbations and reverted to normal or near normal during clinical remission [27].

According to our opinion in our patient there was a greater proximal than distal ulcerative colitis activity. After the treatment with 20 mg prednisone daily for 21 days (before the scintigram) and the treatment with meropenem for 14 days (in case of possible coexistent diverticulitis), the inflammation of sigmoid and rectum retreated and the scintigraphy revealed concentration of gallium-67 exclusively in the ascending colon. Unfortunately, in our case the inflammation of the colon could not be histologically proven because no biopsies were taken from the ascending colon (the colonoscopy was conducted prior to scintigraphy, when there was no indication of inflammation in the ascending colon).

## 4. Conclusions

- Ulcerative colitis activity must be included in the differential diagnosis of FUO in patients with longstanding disease, since fever may present alone, with no other manifestations.
- Nuclear imaging studies are warranted for detecting inflammatory conditions that cannot be diagnosed by other means.
